# Productivity Improvement in Pathology Laboratories Using Motion and Time Study Techniques

**DOI:** 10.5146/tjpath.2023.01610

**Published:** 2024-01-22

**Authors:** Canan Cınar, Afsun Ezel Esatoglu

**Affiliations:** Department of Healthcare Management, Ankara University, Faculty of Health Sciences, Ankara, Turkiye

**Keywords:** Quality, Pathology laboratory, Productivity

## Abstract

***Objective: *** This study aimed to determine the standard time required for the performed job and to examine the standard job critically for productivity improvement in the pathology laboratory.

***Material and Methods:*** In this study that was conducted at a tertiary teaching hospital, observation, fishbone diagram, and flow charts were used to collect the information about the job process. All employees were observed from September 2017 to June 2018. The observations were recorded by video camera in order to overcome the Hawthorne effect. Nine basic procedure steps were followed for productivity improvement.

***Results: *** Within the scope of the study, the jobs of “trimming tissue blocks” and “sectioning of tissue blocks” was selected. The standard time required was 0.19 minutes for “trimming tissue blocks” and 0.34 minutes for “sectioning of tissue blocks”. The procedure steps, named “Removal of tissue block” and “Fixing the block to the device”, were removed from the flow chart to define the improved method. The implementation of the improved method brought in a gain of 11.28 work days per year.

***Conclusion:*** It is obvious that the pathology laboratory needs to take certain measures to improve working conditions and increase efficiency. Our results demonstrate applying the study techniques could reduce the workload and processing time. This study also shows that the study techniques can be applied in the hospital laboratory. Incorporation of all pathology technicians in the change or innovation process will be important in maintaining the achievements.

## INTRODUCTION

Issues such as the structural change of population, developments in science and technology, increased consumption and needs, limited resources, and efficient use of existing resources have caused significant changes in all areas of life (1,2). Recently, there has been growing interest in improving business processes and assessing performance for building sustainability. In this context, the techniques for productivity improvement should be adopted to contribute to the development of employee and business performances (3,4).

A number of work study techniques such as time study and motion study are used to increase productivity and efficiency (5). Time study and motion study have become integrated into a widely accepted method in scientific management. The time study technique is defined as full observation of workers by using a stopwatch to specify the time required to perform certain tasks. The motion study technique seeks out to make processes more efficient by reducing the involved motions (6). The time and motion study techniques, which improve working conditions and offer benefits to improve productivity, are implemented successfully in various industrial and service institutions including healthcare institutions (2,7–9). Application of these techniques in healthcare institutions adds efficiency and quality to service delivery (2,9,10). Reducing errors in healthcare and removing non-value adding activities in processes depend on using these techniques (11). So, these techniques can play a vital role in addressing the issue of planning, employing, and educating the human resources needed in the future to provide effective, efficient, and quality healthcare.

The most important factor that causes labor force problems is the change in the demographic structure of the population. A shortage of labor is nothing new in pathology (12). There has been significant pressure on the service time because of increasing workload and the employment of inadequate pathologists (13). Improvements in the pathology employees’ skills can provide cost reduction and high-quality service (14). Quality means increasing the likelihood of getting the right result in services provided to the patient in healthcare institutions and organizations (15). As in classical industry, patient-related quality control and optimization of working conditions are required in pathology (16).

To ensure quality, the standardization and continuity of the work processes in the pathology laboratory will be possible through time and motion study techniques defining all the work processes. Despite the importance of productivity improvement in pathology, no previous study has employed work study techniques in this field. The purpose of this study was to examine the standard job for productivity improvement in the pathology laboratory through time and motion study techniques. It was thought that this research will guide the application of these techniques for healthcare professionals and researchers in future.

## MATERIALS and METHODS

### Work Environment

The study was conducted in a pathology laboratory that processes more than 30000 cases (about 20000 surgical and 9000 cytology cases) annually, including more than 1200 frozen sections. The laboratory was at a tertiary care teaching hospital in Ankara, Turkey with over 2000 beds, and it was consisted of four separate laboratories: routine histopathology, cytology, immunohistochemistry, and molecular pathology laboratory. About 25 biologists and technicians worked in these laboratories at the time of the study. The laboratory served a normal working day of 09.00 to 17.00 hours Monday to Friday (17). This study has been approved by the Ethics Committee of Ankara University (2018-09-25, 56786525-050.04.04/64696).

### Data Collection

Two instruments were used to capture work activity: direct observation by researcher using a time and motion approach during working hours, and nonstructured observation used to facilitate collection of detailed information. Each employee was observed from September 2017 to June 2018 and on different days between 08:00 a.m. and 17.00 p.m. The observations were recorded by video camera in order to overcome the Hawthorne effect that is known as behavior change in staff to improve performance (18). The results of the observation were recorded by the researcher as descriptive notes. All staff had the right to decline to be observed, and no staff identifiers were used. The video images were deciphered by the researcher according to the time study method.

### The Research Procedure

**Figure 1:**
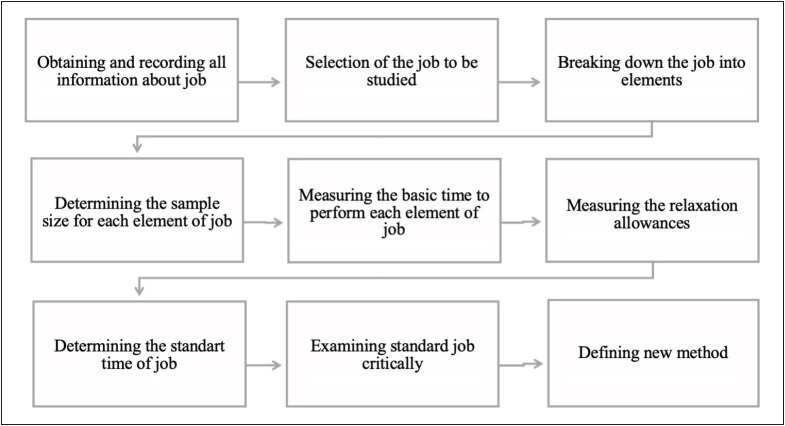
Steps of research procedure.

There was a set procedure that was followed to achieve success in the time and motion study investigation. No motion and time study techniques had previously been performed in this pathology laboratory. Therefore, the time study method was first conducted and the motion study method was then applied to determine the disruptions that occurred. Nine basic steps were identified in this research (3). The following steps were employed in the pathology laboratory (Figure 1).

**Step 1:** The first step in this research procedure was to obtain and record all information about the job. The Fishbone Diagram, which is a visualization tool used for a cause-and-effect analysis for a certain problem/event (19), was used to explore possible causes for a problem in the pathology laboratory.

**Step 2** ***:*** After defining the problems, the job was selected to evaluate improvement points.

**Step 3:** Following step 2, the job was separated into elements to record a complete description of the jobs.

**Step 4:** In order to identify the sample size for each element, the statistical method was applied to present a predetermined confidence level in this study. For the statistical method, a total of ten preliminary readings were first taken. Then, the following equation was applied for the 95% confidence level and a margin of error of 5% (3).


n=\left(\frac{40\sqrt{n1\ast\left(\sum x2\right)-\left(\sum x\right)^2}}{\left(\sum x\right)}\right)^{2\ast}

**Step 5:** For the purpose of this step, the observed time for each selected job element was calculated by using the flyback timing method with a stopwatch. In flyback timing, the hands of the stopwatch are returned to zero at the end of each element and are allowed to start immediately, the time for each element being obtained directly. The calculated observed time for job element was gathered from two qualified technicians who *“have acquired the skill, knowledge and other attributes to carry out the work in hand to satisfactory standards of quantity, quality and safety”* (3). At the same time, the researcher (C.C.) rated the selected technicians’ operating speed through observation. Rating is “*the assessment of worker’s rate of working relative to observer’s concept of the rate corresponding to standard pace*”. The British Standard Scale (0-100 scale) was used in this step to compare between the observed rate of working and the standard rate. In this scale, 0 represents zero activity and 100 the normal rate of working of the standard rate (3). Each element of the job was rated while the technicians performed the relevant job. At the end of step 5, the observed times were adjusted by using the following equation applied to obtain the basic time for each job element.


Basic\ time=\frac{Observed\ time\times Rating}{100}

**Step 6:** In this step, relaxation allowances were calculated to allow the pathology technicians to recover from fatigue, personal needs, contingencies. Since the issues of fatigue or other issues could slow down the rate of working (3), relaxation allowances for fatigue were added to the basic times for each job. The aim of adding relaxation allowances to the basic time is that standard time consists of basic time + relaxation allowance + any allowance for additional job elements (3). To calculate relaxation allowances to compensate, “Tables of Comparative Strains” were taken as reference to allocate a standard time for each element by using the following equation:


Relaxation\ Allowance\ time=\frac{Basic\ time\times Fatigue\ percent}{100}

**Step 7:** For determining the standard time of a job, the researchers calculated the total time that is needed to complete the selected job.

**Step 8: **Critical examination was conducted to apply more effective methods.

**Step 9: **The final step was to define an improved/new method. In this step, it was aimed making the standard job more effective and explain the improved method to the pathology laboratory professionals and management.

### Data Analysis

Times for each job element were calculated with a stopwatch and recorded. The sums of the times of all the elements and activities were transferred into study summary sheets.

## RESULTS

### Step 1. Collecting All Information about the Job

**Figure 2:**
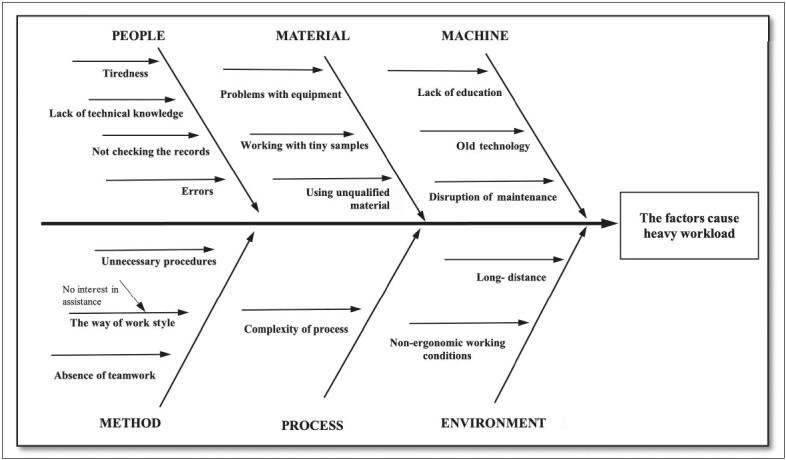
Fishbone diagram.

A fishbone diagram was used to identify the possible causes of a problem and to collect all information about the job in the laboratory. The causes of the problems that led to a heavy workload were identified through observations and brainstorming with technicians. The factors leading to a heavy workload were determined as not checking records, lack of technical knowledge, complexity of the process, and lack of education about the machine in the laboratory. The fishbone diagram revealed that the technicians caused many of the problems in the laboratory (Figure 2).

### Step 2. Selecting the Job

Selecting the job to be studied is the common step in both time and motion study. The standard times are required to analyze efficiency of the original method (3). Therefore, the jobs of *“trimming tissue blocks”* and *“sectioning of tissue blocks”* were selected to determine the standard time and examine the job critically.

### Step 3. Breaking Down the Job into Elements

**Figure 3:**
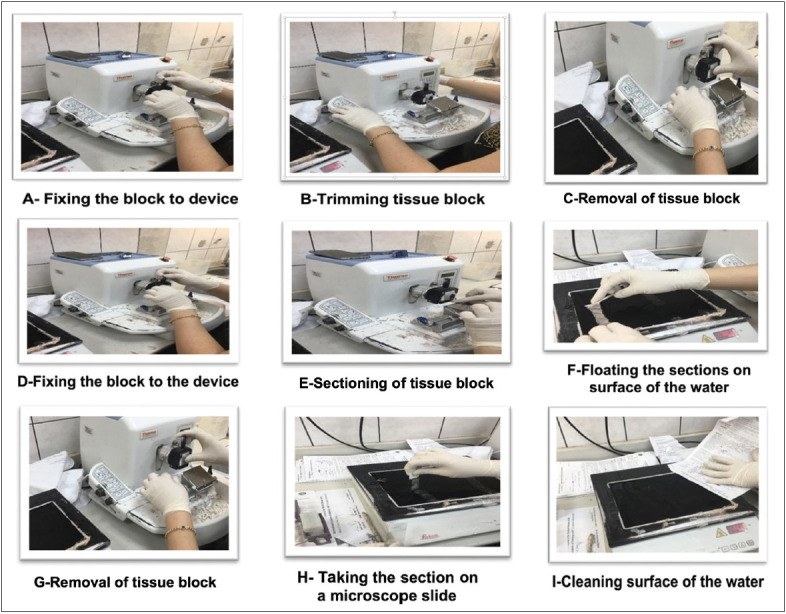
The procedure steps of selected jobs.

After all the information about the job was recorded, the next step was breaking the job down into elements (3). The selected job named *“trimming tissue blocks”* consisted of repeating three stages as: *“A-Fixing the block to the device”*, *“B-Trimming tissue block”* and “*C-Removal of tissue block”* while the other job named “sectioning of tissue blocks” was separated into six phases as: *“D-Fixing the block to the device”*, *“E-Sectioning tissue block”*, *“F-Floating the sections on surface of the water”*, *“G-Removal of tissue block”*, *“H-Taking the section on a microscope slide”* and *“I-Cleaning surface of the water”*. A total of nine job elements were observed within the study (Figure 3).

### Step 4. Determining the Sample Size

The required sample size for the *“trimming tissue blocks”* job was at least 85 observations, and for each element of *“sectioning of tissue blocks” *at least 67 observations were required to ensure that the results are within ±5% accuracy.

### Step 5. Measuring the Basic Time

After the elements were selected and recorded, measuring the basic time step was started. The measurement of the basic times for each element of *“trimming tissue blocks”* based on 85 routine specimens were obtained from two qualified technicians. The basic times for each element of *“trimming tissue blocks”* were as follows: *“A-Fixing the block to the device”* (0.01 min.), *“B-Trimming tissue block”* (0.05 min.) and “*C-Removal of tissue block”* (0.02 min). The total basic time for “trimming tissue blocks” was 0.16 minutes. The measurement of the basic times for each element of *“sectioning of tissue blocks”* were based on 67 routine specimens obtained from two qualified technicians. The basic times for each element of *“sectioning of tissue blocks”* were as follows: *“D-Fixing the block to the device” *(0.02 min.), *“E-Sectioning tissue block”* (0.08 min.),* “F-Floating the sections on surface of the water”* (0.03 min), *“G-Removal of tissue block”* (0.02 min.), “*H-Taking the section on a microscope slide”* (0.06 min.) and *“I-Cleaning surface of the water”* (0.02 min). It was found that the total basic time for* “sectioning of tissue blocks”* was 0.28 minutes (Table 1).

**Table 1:** The standard times for selected jobs.

**The job elements of - **“*trimming of tissue blocks”*		**Basic time**	**Fatigue percent**	**Allowance (min.)**
**Inside job elements**	A-Fixing the block to the device	.019	18	.0034
	B-Trimming tissue block	.052	22	.0114
	C-Removal of tissue block	.022	18	.0040
**Occasional job elements**	Cleaning the device	.012	11	.0013
	Replacing the blade	.005	11	.0006
**Incidental** **shares**	Getting tissue cassettes	.015	5	.0008
	Preparing	.003	5	.0002
	Changing gloves	.036	5	.0018
	Placing the microscope slide in the machine	.003	5	.0002
**Total**		**.167**	**-**	**.0237**
**Standard time **(Total basic time + allowance (min.))				**0.19**
**The job elements -** *“sectioning of tissue blocks”*		**Basic time**	**Fatigue percent**	**Allowance (min.)**
**Inside job elements**	D-Fixing the block to the device	.024	18	.0043
	E-Sectioning tissue block	.081	20	.0162
	F-Floating the sections on surface of the water	.033	18	.0059
	G-Removal of tissue block	.023	18	.0041
**Occasional job elements**	H-Putting the section on a microscope slide	.068	21	.0143
	I-Cleaning surface of the water	.021	18	.0038
	Replacing the blade	.001	11	.0001
	Cleaning the device	.002	11	.0002
**Incidental shares**	Changing gloves	.005	5	.0003
	Preparing	.005	5	.0003
	Taking microscope slide box	.001	5	.0005
	Taking the microscope slide	.015	5	.0008
	Placing the microscope slide in the machine	.008	5	.0004
**Total**		**.287**	**-**	**.0512**
**Standard time **(Total basic time + allowance (min.))				**0.34**

### Step 6. Measuring the Relaxation Allowances

The derivation of the relaxation allowances was based on the data produced from “Tables of Comparative Strains”. Allowance was calculated on the job elements. It can be seen from Table 1 that the total relaxation allowance amounted to 0.02 minutes for *“trimming tissue blocks” *and amounted to 0.05 minutes for *“sectioning of tissue blocks”.*

### Step 7. Determining the Standard Time

By gathering the basic time and relaxation allowance, the standard time required for *“trimming tissue blocks”* was 0.19 minutes, and was 0.34 minutes for *“sectioning of tissue blocks”* (Table 1).

### Step 8. Examining the Standard Jobs

There were fundamental problems in the job processes while examining the standard job critically. When the job processes were thoroughly examined, it was specified that all technicians first trimmed all tissue blocks. In job process, the job steps C and D were repeated by technicians each time. So, the tissue blocks contacted water approximately 30-60 minutes till the phase of sectioning of tissue blocks. This issue caused problems in the diagnosis of the disease during the microscopic examination due to exposition to water for a long time. Also, in the *“sectioning of tissue blocks”* step, the technician should compare the block and microscope slide number to avoid confusion and contribute to better quality results in the output. It was observed that the technicians avoid performing the *“checking the records”* process to reduce processing time.

### Step 9. Defining Improved Method

The improved method is defined in Table 1. To make the standard job more effective, the steps C and D were removed from the flow chart. It would be a gain of 0.05 minutes in the process time and 0.8 meters in the process distance. This would reduce the workload of the technicians and help the pathologist to make the diagnosis on time. So, the *“checking the records”* was added in the *“sectioning of tissue blocks”* step to improve the productivity (Table 2). The implementation of the improved method brought in a gain of a total of 11.28 days (10.42%) in the number of days worked per year (Table 3).

**Table 2:** The flow chart (Original method- Improved method).

**Flow process chart:** ***“*** *Trimming and sectioning of tissue blocks”*						**Original Method**			
**Description**	**Qty.**	**Distance (m)**	**Time (min.)**	**Symbol**					**Remarks**
				○	→	□	>	↓	
On the counter							●		
A-Fixing the block to the device	1	0.3	.022		●				Technician
B-Trimming tissue block	1	-	.063	●					All cases
C-Removal of tissue block	1	0.3	.026		●				Technician
D-Fixing the block to the device	1	0.3	.027		●				“
E-Sectioning tissue block	1	-	.097	●					“
F-Floating the sections on surface of the water	1	0.5	.039		●				“
G-Removal of tissue block	1	0.3	.027		●				“
H-Putting the section on a microscope slide	1	0.3	.082		●				“
I-Cleaning surface of the water	1	-	.025	●					“
**Total**		**2.2**	**.41**	**3**	**6**	**1**	**1**		
**Flow process chart:** ***“*** *Sectioning of tissue blocks”*						**Improved Method**			
On the counter							●		
1-Fixing the block to the device	1	0.3	.022		●				Technician
2-Trimming tissue block	1	-	.063	●					“
3-Sectioning tissue block	1	-	.097	●					“
4-Floating the sections on surface of the water	1	0.5	.039		●				“
5-Removal of tissue block	1	0.3	.027		●				“
6-Checking the records	1	-	.005			●			“
7-Putting the section on a microscope slide	1	0.3	.082		●				“
8-Cleaning surface of the water	1	-	.025	●					“
**Total**		**1.4**	**.36**	**3**	**4**	**1**	**1**		
**Saving**		**0.8**	**.05**	**-**	**2**	**-**	**-**		

**Qty.:** Quantity, **m:** Meter, **min.:** Minute.

**Table 3:** Savings with the improved method.

	**Standard time**	**Number of blocks**	**Total time (day)**	**%**
Original method	0.53 min	194800	119.50	**10.42**
Improved method	0.48 min	194800	108.22	
**Saving**			**11.28**	

## DISCUSSION

This study evaluated the time and motion techniques performed in a pathology laboratory. Although few published studies have examined the productivity by work study techniques in healthcare institutions and organizations, there is no published study about these techniques in the literature of pathology. The aim of work study is to increase productivity in order to enable the transformation of inputs into outputs. The basic graphics and schemes used in work study were communication tools (5). All the information about the job, activities and times were recorded with workflow chart, study summary sheets and time study form in this study**.** These forms and diagrams were thought to offer a broad perspective to management to see how the process works. Identifying duties and responsibilities of laboratory physicians provides information for workload allocation and workforce planning (20).

Taking measures for improving the quality of produced job, developing standardization, early detection of problems, and minimizing the errors have become a part of everyday life (21). Standardization of the work done in the pathology laboratory is an important consideration for improved efficiency and productivity. With the help of job study techniques, standardization can be achieved by identifying the work done in the process and separating the work into its elements (22). In this study, it was found that many problems such as not checking records, lack of technical knowledge, complexity of the process, and lack of education about the machine result as a heavy workload. The previous studies have shown that problems with lack of materials and devices, and problems with equipment supply, cleaning and information are the causes of waste in the pathology laboratory (23,24).

We determined that the jobs of “trimming tissue blocks” and “sectioning of tissue blocks” consisted of nine workflow steps. Within the application of the time study technique in this study, the standard time taken to each job in a pathology laboratory was determined. Our results showed that the standard time of “trimming tissue blocks” was 0.19 minutes and “sectioning of tissue blocks” was 0.34 minutes. A study conducted by Eriguc (25) aimed to find the time spent on “the glove preparation” in the sterilization section by using the time study method and they reported that the total time spent on “glove preparation” as 86.11 seconds. Another study performed in a private hospital based on the time study technique aimed to determine the number of daily scrub and nurses needed for performed operations. The authors have found that the duration of the operation was 2 hours and 53 minutes and the number of operations was an average of 16.4 a day. According to these results, a total of 29 nurses were needed to perform operations (26). In a time study, Sahin et al. (27) investigated a study about workload in terms of nursing activities and the number of nurses required. This study demonstrated that 7 additional nurses were needed in the internal medicine department of a training hospital. In another study conducted by Ozkan and Uydaci (28), work study techniques were used to determine the need for radiology technicians working in hospitals. The study reported that the average examination time for conventional X-rays was 5 minutes, and the current number of radiology technicians should be increased by 17% to meet the standards set.

Time is one of the valuable resources for hospitals. How health care workers spend their time is important for quality and efficiency. Work study helps to classify activities in the delivery of health services (2,11). Removing non-value adding activities in the pathology laboratory contributes to the effective usage of natural resources and staff resource planning (29). Considering the total number of sectioned tissue blocks in 2015 (n=194.800), the time spent on “sectioning of tissue blocks” was 120 days. A study conducted by Melgar et al. (9) used time and method study techniques to determine the time spent on clinical activities by physicians. The study revealed that physicians spent less than half of their time in the clinic with medical assistants. The results of another study revealed that nurses allocated a significant part of their time to “service work” and “personal activities” (27).

The most obvious finding to emerge from this research is that redescribing the job processes in the pathology laboratory though the motion study technique provided process time and distance improvements. The processes and procedures were examined with the motion study technique in this study. Within the motion study method, it is easier to find out the areas of improvement if the process is examined thoroughly. This study determined that there would be a gain of 0.05 minutes in the process time and 0.8 meters in the process distance with the proposed improved method instead of the original method. Another important finding was that the implementation of the improved method in the pathology process with the motion study technique led to a saving of approximately 12 days a year. This result represents important time savings considering that pathology technicians work with small biopsy specimens. In the study conducted by Bircan and Iskender (30), the time study technique was used to evaluate the productivity of the endoscopy procedure in the department of general surgery at a research hospital. This study revealed that the standard time of the endoscopy procedure for the patients where a biopsy was taken to be 15 minutes. However, if the new method was applied in accordance with the proposed methods, the processing time would be reduced. Thus, the number of endoscopies performed in a day could be increased from 9 to 15. Another study conducted by Durur and Akbulut (23) was carried out in a pathology laboratory at a public hospital in Turkey and the authors have claimed that rate of waiting time would be reduced from 73.6% to 69% through eliminating the causes of waste in the pathology laboratory.

The current study focused on the productivity improvement in a part of the pathology laboratory. It is obvious that the pathology laboratory needs to take certain measures to improve working conditions and increase efficiency. In order to ensure quality and standardization, it would be possible to define all the job processes that take place in the pathology laboratory, and to determine the rules and principles. In the creation of the pathology report (15), which is the product of the pathology laboratory, the knowledge and skills of the pathologists are equally important as the laboratory conditions in which the material is prepared (31). For the complete, accurate and timely preparation of the pathology report, the steps during the period until the report reaches the clinical physician must be performed under standard conditions (32). In pathology laboratory studies, the role of technicians is very important in all processes. For this reason, no other laboratory specialty is “technician-dependent” as much as pathology. If the number and quality of technical elements are not sufficient, the risk of erroneous results is quite high (33). Also, our study demonstrates an improved method to access productivity improvement in a pathology laboratory. Further studies should focus on the whole process (including the pre-analytical and post-analytical stage) to present a comprehensive evaluation for problems in pathology. Incorporation of all pathology technicians in the change or innovation process will be important in the continuity of the achievements.

## Conflict of Interest

All authors confirm that there are no conflicts of interest.

## Funding

This study was supported by the Ankara University Scientific Research Projects Coordinatorship.
